# Fighting Antibiotic-Resistant Klebsiella pneumoniae with “Sweet” Immune Targets

**DOI:** 10.1128/mBio.00874-18

**Published:** 2018-05-15

**Authors:** Roberto Adamo, Immaculada Margarit

**Affiliations:** a GSK, Siena, Italy

**Keywords:** antimicrobial resistance, capsular polysaccharide, immune therapy, *Klebsiella pneumoniae*, vaccines

## Abstract

Antibiotics and vaccines have greatly impacted human health in the last century by dramatically reducing the morbidity and mortality associated with infectious diseases. The recent challenge posed by the emergence of multidrug-resistant bacteria could possibly be addressed by novel immune prophylactic and therapeutic approaches. Among the newly threatening pathogens, Klebsiella pneumoniae is particularly worrisome in the nosocomial setting, and its surface polysaccharides are regarded as promising antigen candidates. The majority of *Klebsiella* carbapenem-resistant strains belong to the sequence type 158 (ST258) lineage, with two main clades expressing capsular polysaccharides CPS1 and CPS2. In a recent article, S. D. Kobayashi and colleagues (mBio 9:e00297-18, 2018, https://doi.org/10.1128/mBio.00297-18) show that CPS2-specific IgGs render ST258 clade 2 bacteria more sensitive to human serum and phagocytic killing. E. Diago-Navarro et al. (mBio 9:e00091-18, 2018, https://doi.org/10.1128/mBio.00091-18) generated two murine monoclonal antibodies recognizing distinct glycotopes of CPS2 that presented functional activity against multiple ST258 strains. These complementary studies represent a step toward the control of this dangerous pathogen.

## COMMENTARY

Antimicrobial resistance (AMR) is becoming a serious concern worldwide, leading the World Health Organization (WHO) to a global call for implementing actions to combat the spread of multidrug-resistant (MDR) bacteria (1, 2). The wide usage of broad-spectrum antibiotics in human medicine to treat infections without diagnosis of the specific causative pathogen and in animal husbandry are considered major drivers for bacterial AMR. More than one-third of antibiotic treatments in developed countries occur in health care facilities, and the emergence of infections caused by MDR bacteria in hospitalized patients with underlying morbidity is of particular concern.

MDR is increasing both in Gram-positive pathogens like Streptococcus pneumoniae, Staphylococcus aureus, and Enterococcus spp. and in Gram-negative pathogens like Pseudomonas aeruginosa, Neisseria gonorrhoeae, Escherichia coli, Acinetobacter baumannii, and Klebsiella pneumoniae. Among these pathogens, K. pneumoniae accounted for 8 to 22% of resistance in the United States between 2000 and 2014, with particularly high frequency in the nosocomial setting. K. pneumoniae is a commensal microorganism that can cause chronic urinary tract and soft tissue infections, pneumonia, and sepsis, and it mostly causes disease in immunocompromised subjects. In a recent U.S. surveillance study (3), 25% of infecting K. pneumoniae isolates in the long-term acute care hospital setting were resistant to carbapenems, a powerful group of broad-spectrum beta-lactam antibiotics presently used against penicillin-resistant Gram-negative pathogens. While chromosomally encoded carbapenemases were described in the past, strains bearing plasmid-encoded versions of carbapenemase *bla* genes have become clinically relevant in the last 15 years, raising further concern about lateral transmission among K. pneumoniae and to other enterobacterial species.

Immune prophylactic and therapeutic approaches are expected to play a key role in combating antibiotic-resistant pathogens. By reducing susceptibility to infection, vaccines decrease the necessity for antibiotic treatment and can ultimately limit the environmental pressure leading to the selection of resistant strains. In turn, anti-infective monoclonal antibodies (MAbs) are highly specific therapeutic measures that could be used to block infections in cases of antibiotic failure.

**FIG 1:**
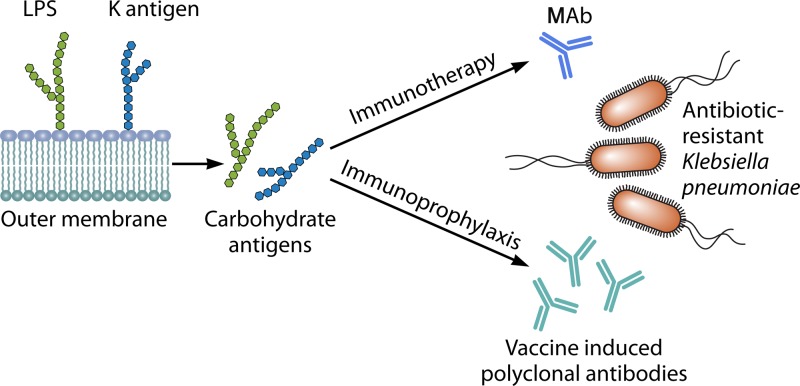
Surface polysaccharides, such as the O antigen and K antigen, from Klebsiella pneumoniae are optimal targets for the development of immunoprophylactic and therapeutic approaches to combat emerging antibiotic-resistant strains, including the hypervirulent ST258.

Glycoconjugate vaccines have been proven highly effective against important human pathogens like Haemophilus influenzae, Neisseria meningitidis, and Streptococcus pneumoniae (4). K. pneumoniae expresses two types of polysaccharide molecules on its surface (Fig. 1), the lipopolysaccharide (LPS) and the capsule, both of which represent promising targets for the development of antimicrobial tools. The LPS is composed of a conserved core oligosaccharide linked to the terminal lipid A portion and to the O antigen, which is encoded in the *rfb* gene cluster and varies in the diverse serotypes. The *cps*-encoded antiphagocytic capsular polysaccharide (CPS) (K antigen) is anchored to the outer membrane through a lipid A tail. Its composition is highly diverse, with at least 77 different variants compared to 9 different O antigens, and additional variants of both have been described by using DNA sequencing approaches (5).

The high structural variability of these surface polysaccharides poses a challenge in the definition of the numerous *Klebsiella* serotypes and also for the development of broadly protective immune system-based preventive and prophylactic tools. Taking in account this high variability, a vaccine composed of 24-valent capsular polysaccharides from epidemiologically relevant serotypes and derived hyperimmune globulins were tested in humans in the late 1980s and 1990s but were not further pursued for development, possibly because of their high complexity (6).

In 2017, Diago-Navarro and colleagues (7) reported the isolation of monoclonal antibodies against the K1 capsular polysaccharide present in up to 81% of hypermucoid hypervirulent strains, the main cause of K. pneumoniae liver abscesses outbreaks in east Asia. These MAbs promoted opsonophagocytic killing, neutrophil extracellular trap (NET) release, and protection against K1 strains in three distinct murine infection models.

In addition to unconjugated capsular polysaccharides, LPS O antigens (O Ags) covalently bound to a variety of carriers, such as iron-regulated cell surface proteins (8), tetanus toxoid (9), and outer membrane proteins (10), were immunogenic in preclinical models, underpinning the potential of this class of sugars as vaccine candidates. The O-Ag variants O1, O2, O3, and O5 could cover a wide range of human infective strains, which would simplify vaccine or therapeutic antibody formulations (5). The use of the O Ag has also been considered for the development of therapeutics to counter MDR K. pneumoniae. In this respect, Szijarto and colleagues just reported a specific humanized antibody directed to the galactan III O antigen, which is expressed in about 83% of the hypervirulent sequence type 258 (ST258) strains (11). Since up to 70% carbapenem-resistant K. pneumoniae strains causing nosocomial infections belong to the multilocus sequence type ST258, this lineage represents an important target for vaccines and therapeutics (12). The anti-galactan III MAb was capable of neutralizing the LPS endotoxin activity and mediating opsonophagocytic and serum killing of strains belonging to this clonal group.

Two recent *mBio* articles report the results of two teams that concentrated on the capsular polysaccharide as a potential target of protective antibodies against carbapenem-resistant ST258. There are two main clades of K. pneumoniae ST258 which differ mainly in a 215-kb region that comprises genes involved in capsular polysaccharide biosynthesis, resulting in the expression of two different capsular types, CPS1 and CPS2.

Kobayashi and colleagues (13) confirmed the contribution of CPS2 in the resistance of an ST258 strain to complement-mediated killing in human serum and in neutrophil escape. They generated polyclonal sera by immunizing rabbits with multiple doses of CPS1 or CPS2 and investigated the capacity of these sera to enhance complement-mediated bactericidal activity of clade 1 and 2 strains and their ingestion and killing by human polymorphonuclear leukocyte (PMN) phagocytes. In the presence of anti-CPS2 polyclonal sera and purified IgG, clade 2 bacteria became more sensitive to human serum, and their phagocytosis increased. The same effect was not observed for anti-CPS1 on a clade 1 strain that presented higher background susceptibility to human phagocytes.

Diago-Navarro and colleagues (14) confirmed and expanded these findings by two newly generated mouse monoclonal antibodies against clade 2 CPS. The two IgG3 MAbs differed by only three amino acids, yet glycoarray experiments indicated that the MAbs recognized distinct glycotopes. Both antibodies showed functional activity against multiple ST258 strains, mediating partial prevention of biofilm formation, complement deposition, intracellular bacterial killing, enhanced formation of bactericidal NETs by human neutrophils, as well as reduced bacterial dissemination in intratracheally infected mice. The results of a competition enzyme-linked immunosorbent assay (ELISA) suggested a cooperative effect, as binding of one of the MAbs to CPS2 was enhanced in the presence of the second, although cooperative functional activity was not investigated.

The possible impact that emergence of new MDR K. pneumoniae epidemic clones by lateral transfer and recombination will have on clinical disease and the efficacy of polysaccharide-based immune targets remains to be seen. Nevertheless, these studies open the path to antibody-based immunotherapies to combat the currently threatening K. pneumoniae lineages (Fig. 1). Importantly, these tools may be applied to future vaccine antigen combinations and MAb cocktails against both capsular and LPS targets, offering wider coverage against the threat of MDR K. pneumoniae.
